# Patient-Reported Outcome Measures for assessing functioning, disability, and health of people with spinal cord injury – a scoping review

**DOI:** 10.1080/10790268.2025.2477364

**Published:** 2025-05-05

**Authors:** Eelis Nuuttila, Sanna-Mari Saarimäki, Arto J. Hautala, Sinikka Hiekkala

**Affiliations:** 1 Faculty of Sport and Health Sciences, University of Jyväskylä, Jyväskylä, Finland; 2 The Finnish Association of People with Physical Disabilities, Helsinki, Finland

**Keywords:** Spinal cord injury, Patient-Reported Outcome Measure, Psychometrics, ICF, Scoping review

## Abstract

**Objective:**

The purpose of this study was to identify the most frequently used Patient-Reported Outcome Measures (PROM) for people with spinal cord injury (SCI) and to explore how they assess function, disability, and health in the framework of the International Classification of Functioning, Disability and Health (ICF). Additionally, the psychometric properties of the identified PROMs were investigated.

**Design:**

Scoping review.

**Methods:**

MEDLINE (Ovid) and CINAHL (EBSCO) electronic databases were searched for PROMs that assess functioning, disability, and health in people with SCI. Eligible PROMs were linked to the ICF and further screened based on how thoroughly they covered the ICF categories of the Brief ICF Core Sets for people with SCI. Finally, a search was performed to investigate the psychometric properties of selected PROMs.

**Results:**

Of the most frequently applied PROMs, 21 were linked to the ICF. Items of the Craig Hospital Inventory of Environmental Factors (CHIEF), the Nottwil Environmental Factors Inventory Short Form (NEFI-SF), the Spinal Cord Injury Secondary Conditions Scale (SCI-SCS), and the Spinal Cord Independence Measure – Self Report (SCIM-SR) covered the constructs of the Brief ICF Core Sets most comprehensively, and their psychometric properties were assessed. The psychometric properties of the SCIM-SR had been examined the most thoroughly, and its validity and reliability were acceptable.

**Conclusions:**

Sufficient psychometric properties encourage using the SCIM-SR to assess functioning in daily activities of people with SCI whereas CHIEF, NEFI-SF, and SCI-SCS measure meaningful constructs but need more psychometric research.

## Introduction

Spinal cord injury (SCI) is a life-altering condition with serious physical, psychosocial, and economic effects on those affected by the injury and on a larger societal scale [1, 2]. Neurological damage caused by the SCI interferes with travel of motor and sensory information between brain and target tissue below the level of injury [3]. In addition to the loss of motor and sensory function, SCI can cause a variety of secondary health conditions such as spasticity, chronic pain, sexual dysfunctions, bladder and bowel dysfunctions, and sleep problems [4]. Comorbidities, for example high blood pressure, back problems, high cholesterol, diabetes, substance use, and psychiatric conditions, are also prevalent in people with SCI [5, 6].

Appropriate medical and rehabilitation interventions can prevent complications associated with SCI and can contribute toward achieving a fulfilling and prospective life [3]. Assessment of function is an integral part of the rehabilitation process [7] and evaluating impact of treatment [8]. A Patient-Reported Outcome Measure (PROM) is a subjective measure of a person’s conditions where any outcomes are evaluated by the person directly without input from a clinician or anyone else [9]. PROMs can assess various domains of health status, such as experience of disability, symptoms and quality of life. Through PROMs, it is possible to gain a more complete understanding from the responses of the people themselves [10]. Obtaining information from the perspective of persons living with SCI is valuable as injury severity is not the only thing affecting the experience and perception of health [11, 12].

In a clinical setting, the International Classification of Functioning, Disability and Health (ICF) is designed to assess health states and evaluate rehabilitation outcomes [13]. Through the ICF, health and functioning are considered in a holistic manner as the ICF defines health, functioning and disability in the context of Body Functions and Structures, Activities and Participation, Environmental Factors, and Personal Factors, which all influence and interact with each other [14]. The ICF provides a standard language and framework for describing health and health-related states [15]. However, since the ICF is designed to record a wide range of health-related information and is intended to be used across multiple sectors, it should, in a practical sense, be tailored for specific applications. Generally agreed on lists of meaningful ICF categories for a specific condition can serve as a Core Set, which should be rated for every all individuals with the condition [13, 16].

ICF Core Sets were designed to capsulize ICF categories relevant to a specific health status or disability. Comprehensive ICF Core Sets can guide multidisciplinary assessment of patients in a particular condition. Brief ICF Core Sets include as few categories as possible to be practical but still as many as necessary to comprehensively describe a typical spectrum of issues in a set population. A Brief Core Set can serve as a minimum data set to be reported in all clinical studies to describe the burden of disease in a comparable way across studies [16]. Such Core Sets exist for the SCI in different clinical applications [17–20]. PROMs can be linked to the ICF to reveal the constructs and areas that the outcome measures cover and so reaffirm they measure the desired constructs in a selected condition [21, 22].

A wide variety of outcome measures are available for assessing the functioning of people with SCI, but there is a lack of consensus on what outcome measures, let alone PROMs, should be used [23–25]. Adequate psychometric properties, such as validity, reliability, and responsiveness, should be assessed when selecting an outcome measure and they should be assessed with the population of interest in mind [26]. Generic outcome measures can be used for people with SCI, but they may not effectively capture relevant constructs or may lack sufficient psychometric research within the SCI population [27, 28]. The purpose of this scoping review was (1) to summarize the most frequently used PROMs available for assessing various aspects of health functioning, disability, and health in people with SCI; (2) to explore how the PROMs link to the ICF; and (3) to investigate the psychometric properties of those outcome measures that cover the Brief ICF Core Sets for people with SCI most thoroughly.

## Methods

This study adhered to the scoping review methodology in the Joanna Briggs Institute guidelines [29]. A scoping review was chosen as the most appropriate methodology for identifying possible gaps in existing literature and summarizing and disseminating the research findings [30]. The Preferred Reporting Items for Systematic reviews and Meta-Analyses extension for Scoping Reviews (PRISMA-ScR) reporting guidelines were followed [31].

The literature search process and study selection were done in two different stages. In stage one, first a systematic search was performed to find articles reporting the use of PROMs in a population of people with SCI. Then, articles were screened, and the identified PROMs were charted. PROMs utilized in ten or more different research articles were linked to the ICF, following the framework of Cieza *et al*. [22]. The cut-off point of ten articles was chosen to help in screening the most frequently used PROMs out of the hundreds available. Additionally, only freely available PROMs were chosen, as the results of this scoping review are to be used to form recommendations for general clinical use. If both a brief and a full version of a single PROM appeared in the most frequently used PROMs, the full version was linked to the ICF. The ICF linking was done independently by two researchers (EN, SH).

In stage two, after linking all items of the selected PROMs to the ICF, the ICF-linked PROMs were screened based on how thoroughly they covered the ICF categories of the acute and chronic Brief ICF Core Sets for people with SCI [18, 20]. Then, based on how thoroughly they covered those ICF categories, the research group unanimously chose the PROMs whose psychometric properties were explored through a systematic search. A combination of both the acute and the chronic Brief ICF Core Sets was chosen to ensure a sufficient number of relevant categories for comparisons and to keep the number of categories still practical [16]. A protocol for this scoping review was registered on Open Science Framework (https://osf.io/5czq9).

### Eligibility criteria

In stage one, the following criteria were used to include articles to map the usage of PROMs: (1) the frequency of people with SCI was reported; (2) a standardized PROM was used; and (3) a full-text article in English was available. The exclusion criteria were: (1) SCI population of a solely congenital origin; (2) the PROM had only one item; (3) the PROM was used as a telephone or face-to-face interview tool; (4) only selected items of the PROM were used, items were added, or an alteration was made to the phrasing of the items; and (5) the found literature were books, book chapters, theses, editorials, or opinion pieces.

In stage two, when the psychometric properties of the selected PROMs were investigated, the inclusion criteria were: (1) at least 50% of the sample were people with SCI; (2) a peer-reviewed journal article assessing psychometric properties of a selected PROM was found; and (3) a full-text article in English was available.

### Information sources

Two electronic databases were used to search for relevant publications: MEDLINE (Ovid) and CINAHL (Ebsco). The search for relevant PROMs was conducted on the 14th of December 2022, and the complementary search of psychometric properties was performed through the 3rd and 4th of August 2023. Publication dates were not limited. The reference lists of the scoping and systematic reviews identified in the screening process were searched.

### Search

The initial search strategy was developed by two researchers (EN, SH) and piloted by one of the researchers (EN). Based on the results of the pilot search, the subject headings and keywords were further improved. In the first search of relevant PROMs, search terms associated with SCI such as “spinal cord injury”, “spinal injuries”, and “tetraplegia” were used in combination with search terms associated with PROMs such as “Patient-Reported Outcome Measure”, “survey”, and “questionnaire” (supplementary table 1). The second stage of the search used a combination of terms related to validity, reliability, responsiveness, and interpretability in addition to the identified PROM and its abbreviations. Search term selection was guided by The Guide for the Evaluation of Functional Outcome Measures [32] (supplementary table 2).

### Selection of sources of evidence

Search results from the electronic databases were imported into Zotero review management software where duplicates were removed. A random sample of 25 articles was used to pilot the screening process, and the formal screening was initiated once the agreed level of consensus (deemed as > 75%) was achieved. Two researchers (EN, SH) screened the titles and abstracts for the full-text inclusion and conducted the full-text screenings as well. All discrepancies were solved by discussion. The Consensus-based Standards for the selection of health Measurement Instruments (COSMIN) and The Guide for Evaluation of Functional Outcome Measures were utilized for the quality appraisal of the psychometric measurement properties [32, 33]. Two independent researchers (EN, SMS) screened the abstracts, titles, and full texts of the articles covering the psychometric properties of the selected PROMs. Differing opinions were resolved by discussion or by a third reviewer (SH).

### Data charting process

Data were charted in tables in Microsoft Excel. In stage one, the identified PROMs, the titles of the publications where the PROMs were identified, names of the authors, and the publication year were mapped. In stage two, the following data were extracted: name and description of the outcome measure, information about the psychometric studies, title, authors, year of publication, and reported psychometric properties. The data extraction was performed by one of the researchers (EN) except for the psychometric properties of the NEFI-SF (SMS).

## Results

### Search results

**Figure 1:**
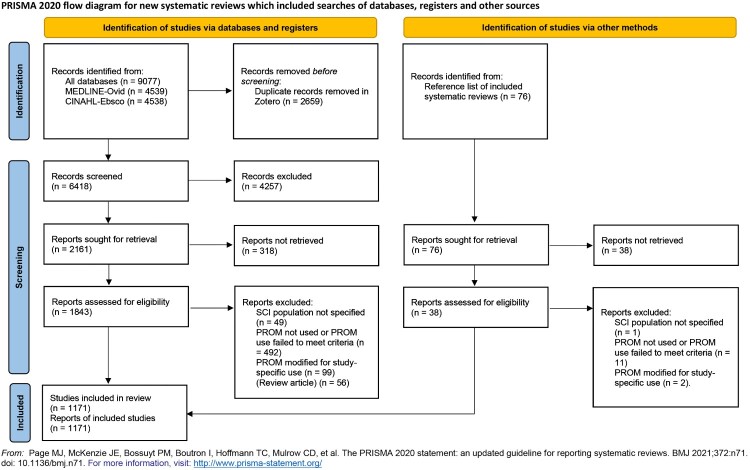
PRISMA flow diagram of article search.

Electronic database searches yielded a total of 9077 identified records, and after the removal of duplicates, 6418 titles and abstracts remained for screening (Fig. 1). The full texts of 1843 articles were assessed, and 1147 were included in the scoping review. In addition, 76 articles were identified from the reference lists of the included review articles. Of these, 24 articles were chosen, making the total number of articles included in the scoping review 1171.

### PROM characteristics

Of the included 1171 articles, a total of 595 distinct PROMs were identified (supplementary table 3). A wide variety of PROMs were found, but a small minority of them appeared in several different articles. In accordance with the inclusion criteria, 55 different PROMs had been applied in ten or more different research articles. Of those PROMs, 19 were not freely available. A substantial portion of them were designed to measure aspects of mental health or bladder and bowel symptoms such as Hospital Anxiety and Depression Scale (HADS) [34], The Qualiveen Questionnaire [35] and Beck Depression Inventory (BDI) [36]. Ten PROMs could not be linked to the ICF. For example, the Appraisal of Life Events Scale is an index of the threat, challenge, and loss appraisal dimensions and an adjective check list that can be used to assess appraisals of retrospectively recalled stressful events as well as ongoing stressful encounters [37]. Five of the outcome measures were a shortened version of a previously identified PROM.

A descriptive summary and ICF linking were done for 21 PROMs (Table 1). Half (*n* = 11) of the most frequently utilized PROMs were originally developed prior to the year 2000. The most recently developed PROMs were The Spinal Cord Independence Measure – Self Report (SCIM-SR) [38] (2013), the Utrecht Scale for Evaluation of Rehabilitation – Participation (USER-Participation) [39] (2012), and the Nottwil Environmental Factors Inventory Short Form (NEFI-SF) [40] (2015). Of the 21 most frequent PROMs, five (the NEFI-SF, the SCIM-SR, the Moorong Self-Efficacy Scale (MSES) [41], the Spinal Cord Injury Secondary Conditions Scale (SCI-SCS) [42], and the International Spinal Cord Injury Pain Basic Data Set (ISCIPBDS) [43]) were specifically designed to be used in people with SCI.

**Table 1:** Characteristics of the most frequently used Patient-Reported Outcome Measures (PROMs) for people with spinal cord injury.

PROM, (Short forms)	Brief description of the PROM purpose	Frequency in included articles, *n* (short forms)
SF-36, (SF-12)	General multipurpose health survey [55]	101, (34)
PHQ-9	To assist in diagnosis of depression [44]	88
CHART, (CHART-SF)	To assess WHO dimensions of handicap [52]	40, (17)
WHOQOL -BREF	To assess domains of quality of life [56]	53
LISAT-11, LISAT-9	To measure life satisfaction [53]	14, (31)
CESD, (CESD-10)	To measure depressive symptoms in general population [45]	34, (10)
MPQ, (SF-MPQ)	To assess intensity and quality of pain [49]	18, (17)
SCIM-SR	To measure functioning in activities of daily living in people with SCI [38]	26
MSES	To measure self-efficacy in people with SCI [41]	24
SCI-SCS	To assess secondary health conditions of people with SCI [42]	22
PSS	To measure the degree to which situations in one's life are appraised as stressful [47]	20
FSS	To measure the severity of fatigue [57]	17
IPA	To measure several aspects of participation and autonomy [54]	15
USER-Participation	To measure participation in daily life distributed between frequency, restrictions, and satisfaction [39]	14
MSPSS	To assess an individual’s perception of the social support [50]	14
DASS-21	To measure the three related negative emotional states of depression, anxiety, and tension/stress [46]	12
CHIEF	To measure the frequency and magnitude of environmental barriers reported by individuals [58]	11
DN4	To identify neuropathic pain [48]	11
ISCIPBDS	To assess pain severity, physical and emotional function, and the impact of pain [43]	10
NEFI-SF	To measure perceived negative impact of environmental barriers on participation and daily life in people with SCI [40]	10
QUEST	To measure satisfaction with assistive technology [51]	10

Note: CESD/CESD-10, Center of Epidemiological Studies Depression Scale; CHART, Craig Handicap Assessment and Reporting Technique; CHART-SF, Craig Handicap Assessment and Reporting Technique Short Form; CHIEF, Craig Hospital Inventory of Environmental Factors; DASS-21, Depression Anxiety and Stress Scale Short Form; DN4, Douleur Neuropathique 4; FSS, The Fatigue Severity Scale; IPA, Impact on Participation and Autonomy; ISCIPBDS, International Spinal cord Injury Pain Basic Data Set; LISAT-9/LISAT-11, Life Satisfaction Questionnaire; MPQ, McGill Pain Questionnaire; MSES, Moorong Self Efficacy Scale; MSPSS, Multidimensional Scale of Perceived Social Support; NEFI-SF, Nottwill Environmental Factors Inventory Short Form; PHQ-9, 9-item The Patient Health Questionnaire; PSS, Perceived Stress Scale; QUEST, The Quebec User Evaluation of Satisfaction with Assistive Technology; SCI, Spinal cord injury; SCI-SCS, Spinal Cord Injury-Secondary Conditions Scale; SCIM-SR, Spinal Cord Independence Measure Self Report; SF-12, 12-Item Short Form Survey, SF-36, 36-Item Short Form Survey; SF-MPQ, McGill Pain Questionnaire Short Form; USER-Participation, Utrecht Scale for Evaluation of Rehabilitation Participation; WHOQoL-Bref, The World Health Organization Quality of Life.

### Linking the PROM to the ICF

Eligible PROMs were first linked to the ICF and then analyzed regarding how they covered constructs according to the acute and chronic Brief ICF Core Sets developed for people with SCI [18, 20]. PROMs designed to assess distinct concepts were linked to a single ICF category – either the b152 “Emotional functions” or the b280 “Sensation of pain” (Table 2). They included: the Patient Health Questionnaire – 9 (PHQ-9) [44], the Center for Epidemiological Studies-Depression (CES-D) [45], and the Depression, Anxiety and Stress Scale – 21 (DASS-21) [46] for measuring depressive and/or anxiety symptoms; the Perceived Stress Scale (PSS) [47] for stress symptoms; and the Douleur Neuropathique 4 (DN4) [48], the ISCIPBDS, and the McGill Pain Questionnaire (MPQ) [49] for measuring pain. The Multidimensional Scale of Perceived Social Support (MSPSS) [50], which is designed to measure perceived social support, was linked to the e310 “Immediate family” category. The Quebec User Evaluation of Satisfaction with Assistive Technology (QUEST) [51] was linked to the category e355 “Health professionals”.

**Table 2:** Patient-Reported Outcome Measures (PROMs) linked to the brief acute and chronic ICF Core Sets for people with spinal cord injury.

ICF COMPONENT AND CATEGORIES	PROM																		
	CESD	CHART	CHIEF	DASS-21	DN4	IPA	ISCIPBDS	LISAT-9	MSES	MSPSS	NEFI-SF	PHQ-9	PSS	QUEST	SCI-SCS	SCIM-SR	SF-36	SF-MPQ	WHOQoL-Bref
**BODY FUNCTIONS**																			
b730 Muscle power functions																			
b620 Urination functions															x	x			
b280 Sensation of pain					x		x								x		x	x	x
b525 Defecation functions									x						x	x			
b640 Sexual functions								x							x				
b810 Protective functions of the skin															x				
b735 Muscle tone functions															x				
b710 Mobility of joint functions															x				
b152 Emotional functions	x			x								x	x				x		x
b440 Respiration functions															x	x			
**BODY STRUCTURES**																			
s120 Spinal cord and related structures																			
s610 Structure of the urinary system																			
s810 Structure of areas of the skin																			
s430 Structure of respiratory system																			
**ACTIVITIES AND PARTICIPATION**																			
d530 Toileting						x										x			
d420 Transferring oneself																x			
d230 Carrying out daily routine																			x
d465 Moving around using equipment																x			
d410 Changing basic body position																x	x		
d445 Hand and arm use																			
d470 Using transportation		x																	x
d455 Moving around						x										x	x		
d520 Caring for body parts																x			
d550 Eating						x										x			
d240 Handling stress and other physical demands																			
d450 Walking																	x		
d510 Washing oneself						x			x							x	x		
d540 Dressing						x										x	x		x
d560 Drinking						x										x			
**ENVIRONMENTAL FACTORS**																			
e310 Immediate family			x						x	x									x
e120 Products and technology for personal indoor and outdoor mobility and transportation			x								x								
e115 Products and technology for personal use in daily living			x								x					x			
e150 Design, construction and building products and technology of buildings for public use			x								x								
e155 Design, construction and building products and technology of buildings for private use			x								x								
e110 Products or substances for personal consumption											x								
e355 Health professionals											x			x					
e340 Personal care providers and personal assistants											x								
e580 Health services, systems and policies																			x

Note: In the ICF b refers to Body Functions, s to Body Structure, d to Activities and Participation and e to Environmental Factors. ICF, International Classification of Functioning, Disability and Health; CESD, Center of epidemiological studies depression scale; CHART, Craig Handicap Assessment and Reporting Technique; CHIEF, Craig Hospital Inventory of Environmental Factors; DASS-21, Depression Anxiety and Stress Scale Short Form; DN4, Douleur Neuropathique 4; IPA, Impact on Participation and Autonomy; ISCIPBDS, International Spinal Cord Injury Pain Basic Data Set; LISAT-9, Life Satisfaction Questionnaire; MSES, Moorong Self Efficacy Scale; MSPSS, Multidimensional Scale of Perceived Social Support; NEFI-SF, Nottwill Environmental Factors Inventory Short Form; PHQ-9, 9-item The Patient Health Questionnaire; PSS, Perceived Stress Scale; QUEST, The Quebec User Evaluation of Satisfaction with Assistive Technology; SCI-SCS, Spinal Cord Injury-Secondary Conditions Scale; SCIM-SR, Spinal Cord Independence Measure Self Report; SF-36, 36-Item Short Form Survey; SF-MPQ, McGill Pain Questionnaire Short Form; WHOQoL-Bref, The World Health Organization Quality of Life.

The Craig Handicap Assessment and Reporting Technique (CHART) [52] and the Life Satisfaction Questionnaire (LISAT-11) [53] are PROMs measuring general life satisfaction or participation. Each of them covers a single ICF category from the combined Brief ICF Core Sets for people with SCI. It was possible to link the CHART to the d470 “Using transportation” category and the LISAT-11 to the b640 “Sexual functions” category.

A number of items of the Impact on Participation and Autonomy (IPA) [54], the 36-Item Short Form Survey (SF-36) [55], and the World Health Organization Quality of Life (WHOQoL-Bref) [56] were linked to several categories of the Brief ICF Core Sets, mainly in the “Activities and Participation” domain. Items from the USER-Participation [39] and the Fatigue severity Scale (FSS) [57] were linked to the ICF but did not cover any of the categories of the Brief ICF Core Sets. For example, items from the FSS were linked to b4552 “Fatiguability”, which is not present at the Brief ICF Core Sets.

None of the most frequently used PROMs could be linked to any of the categories in the “Body Structures” domain, nor to the categories b730 “Muscle power functions”, d445 “Hand and arm use”, d240 “Handling stress and other physical demands”, or e580 “Health services, systems and policies”.

The SCI-SCS covered the most categories in the “Body Functions” domain, the SCIM-SR the most in the “Activities and Participation” domain, and the CHIEF [58] and the NEFI-SF the most in the “Environmental Factors” domain. Those four PROMs were then unanimously selected by the research group to go through a systematic search to explore their psychometric properties.

### Psychometric properties of Patient-Reported Outcome Measures

A complementary search of the psychometric properties of the selected PROMs yielded a total of 42 articles. After the title, abstract, and full-text screenings, 16 articles were included in the scoping review. Of the 16 articles, one addressed the CHIEF, one the NEFI-SF, four the SCI-SCS, and 10 the SCIM-SR. A summary of the articles and extracted psychometric properties of each PROM are presented in Table 3.

**Table 3:** Summary of the psychometric studies of the selected Patient-Reported Outcome Measures (PROMs) for people with spinal cord injury.

PROM	Description	Scoring	Authors, year and title	Participants	Measurement properties
**CHIEF**	25-item self-report instrument to measure impact of environmental barriers. Covers domains of accessibility, accommodation, resource availability, social support, and equality.	Each item has a frequency score (0–4) and a magnitude score (0–2). An impact score (0–8) is gained by multiplying the former two. Total scores across the 25 items are calculated as the average frequency-magnitude product score. A higher score represents increased environmental barriers.	Soni, S., Walia, S., & Noohu, M. M. (2016). Hindi translation and evaluation of psychometric properties of Craig Hospital Inventory of Environmental Factors instrument in spinal cord injury subjects. Journal of Neurosciences in Rural Practice, 7(01), 13-22.	*n* = 30	Content validity
** **				Complete or incomplete SCI	Internal consistency
** **				Time since injury > 1 year	Interpretability
** **				Mean age 31.67 (SD 10.09)	Measurement error
** **				Tetraplegia *n* = 14, paraplegia *n* = 16	Test-retest reliability
** **				AIS A 7, AIS B 13, AIS C 7, AIS D 3	
**NEFI-SF**	14-item self-report instrument founded on the ICF Core Sets for SCI. Items cover potential environmental barriers, such as public and home access, climate, communication devices, finances, medical supplies, personal care assistance, political decisions, social attitudes, and transport. Designed to be used in population-based studies to measure the environmental barriers that made life harder for people with SCI within the last four weeks.	Respondents assess the negative influence of each environmental barrier on their lives within the past 4 weeks. The answer options are “no influence / not applicable” (0), “made my life a little harder” (1), and “made my life a lot harder” (2). High NEFI-SF total scores (range 0–28) indicate more perceived restrictions on participation due to environmental barriers.	Ballert, C. S., Post, M. W., Brinkhof, M. W., & Reinhardt, J. D. (2015). Psychometric Properties of the Nottwil Environmental Factors Inventory Short Form. Archives of physical medicine and rehabilitation, 96(2), 233-240.	*n* = 1549 Traumatic or nontraumatic SCI Time since injury 14.24 (IQR 7.0–25.3) years Median age 55.36 (IQR 44.9–66.1) Traumatic 72%, non-traumatic 28% Tetraplegia 31%, paraplegia 69% Complete 42%, incomplete 58%	Construct validity Cross-cultural validity Floor and ceiling effect Internal consistency Structural validity
** **					
**SCI-SCS**	Self-reported health measure scale for persons with SCI. Comprises 16 symptoms or conditions of which the respondents are asked to evaluate their health for the last 3 months.	Secondary health conditions are self-rated on a scale of 0 to 3: (0) "not experienced in the last 3 months or is an insignificant problem", (1) "mild or infrequent problem", (2) "moderate or occasional problem", (3) "significant or chronic problem". The maximum score is 48 and a higher score represents more experienced health problems.	Arora, M., Harvey, L. A., Lavrencic, L., Bowden, J. L., Nier, L., Glinsky, J. V., … & Cameron, I. D. (2016). A telephone-based version of the spinal cord injury–secondary conditions scale: a reliability and validity study. Spinal Cord, 54(5), 402-405.	*n* = 40 Complete or incomplete traumatic or non-traumatic SCI Time since injury > 3 months Median age 54 (range 48–63) AIS A 67%, AIS B 28%, AIS C 5% Injury level C2–T12	Convergent validity Inter-rater reliability
** **			Jørgensen, V., von Rosen, P., & Butler Forslund, E. (2021). Considerations on the psychometric properties and validity of the Spinal Cord Injury Secondary Conditons Scale. Spinal Cord, 59(8), 894-901.	*n* = 224 Median time since injury 15.0 (IQR 6.0) years Mean age 49.5 (SD 14.9) AIS A 45%, AIS B 14%, AIS C 9%, AIS D 32% Cervical 51%, T 1–6 15%, T7–12 26%, lumbar 8%	Construct validity Floor and ceiling effect Hypothesis testing Internal consistency
** **			Kalpakjian, C. Z., Scelza, W. M., Forchheimer, M. B., & Toussaint, L. L. (2007). Preliminary reliability and validity of a spinal cord injury secondary conditions scale. The journal of spinal cord medicine, 30(2), 131-139.	*n* = 65 Complete or incomplete traumatic or non-traumatic SCI Time since injury ≥ 1 year Mean age 43.8 (SD 13.4) Paraplegia incomplete 11.8%, paraplegia complete 39.7%, tetraplegia incomplete 20.6%, tetraplegia complete 23.5% Injury level ≤ C5	Convergent validity Internal consistency Test-retest reliability
** **			Conti, A., Clari, M., Arese, S., Bandini, B., Cavallaro, L., Mozzone, S., … & Campagna, S. (2020). Validation and psychometric evaluation of the Italian version of the Spinal Cord Injury Secondary Conditions Scale. Spinal cord, 58(4), 496-503.	*n* = 156 Complete or incomplete traumatic or non-traumatic SCI Mean age 50,17 (SD 14,33) AIS A 37,4 %; AIS B, C or D 62,6 %	Concurrent validity Construct validity Face validity Floor and ceiling effect Internal consistency Test-retest reliability
**SCIM-SR**	Self-reported measure to assess functionality of a person with a spinal cord injury. Comprises of 17 items to evaluate perfromance of physical tasks such as eating and drinking, dressing, self-care, and mobility.	17 items divided into 3 sub-scales: self-care, respiration and sphincter management, and mobility. Self-care (score range 0–20), respiration and sphincter management (score range 0–40) and mobility (score range 0–40). Total score ranges between 0 and 100. The higher the score, the better the individual’s level of independent functioning.	Fekete, C., Eriks-Hoogland, I., Baumberger, M., Catz, A., Itzkovich, M., Lüthi, H., … & Brinkhof, M. W. G. (2013). Development and validation of a self-report version of the Spinal Cord Independence Measure (SCIM III). Spinal cord, 51(1), 40-47.	*n* = 99 Traumatic or non-traumatic SCI Time since injury ≥ 1 month Median age 48 (IQR 35.0–64.0) Paraplegia 53.4%, complete lesion 43.8%	Criterion validity
** **			Takeuchi, S., Uemura, O., Unai, K., & Liu, M. (2021). Adaptation and validation of the Japanese version of the spinal cord Independence measure (SCIM III) self-report. Spinal Cord, 59(10), 1096-1103.	*n* = 100 Complete or incomplete traumatic or non-traumatic SCI. Median age 63 (IQR 52–70) Cervical *n* = 65, thoracic *n* = 29, lumbar and cauda equina *n* = 6 Complete injury *n* = 32, incomplete injury *n* = 68	Concurrent validity Internal consistency
** **			Wilartratsami, S., Luksanapruksa, P., Santipas, B., Thanasomboonpan, N., Kulprasutdilok, P., Chavasiri, S., & Chavasiri, C. (2021). Cross-cultural adaptation and psychometric testing of the Thai version of the spinal cord Independence measure III—self report. Spinal Cord, 59(3), 291-297.	*n* = 32 Traumatic or non-traumatic SCI Time since injury ≥ 3 months Mean age 44.97 (SD 20.31) Injury level C1–C4 9%, C5–C8 47%, upper thoracic 28%, lower thoracic 16% Traumatic 72%, non-traumatic 28% AIS A 61%, AIS B 6%, AIS C 10%, AIS D 23%	Concurrent validity Floor and ceiling effect Internal consistency Measurement error Test-retest reliability
** **			Prodinger, B., Ballert, C. S., Brinkhof, M. W., Tennant, A., & Post, M. W. (2016). Metric properties of the Spinal Cord Independence Measure-Self Report in a community survey. Journal of rehabilitation medicine, 48(2), 149-164.	*n* = 1530 Complete or incomplete traumatic or non-traumatic SCI Mean age 52.33 (SD 14.77) Non-traumatic incomplete (16.6%), complete (5.1%) tetraplegia. Non-traumatic incomplete (35.6%), complete (13.3%) paraplegia	Construct validity Floor and ceiling effect
** **			Khatri, P., Prasertsukdee, S., & Suttiwong, J. (2022). Reliability of the Nepali Version of the Spinal Cord Independence Measure Self-Report. Rehabilitation Research and Practice, 2022.	*n* = 45 Traumatic or non-traumatic SCI Time since injury ≥ 1 month Mean age 29.6 (SD 8.9, range 18–59) Tetraplegia 17.8%, paraplegia 82.2% AIS A 77.8%, AIS B 13.3%, AIS C 8.9%	Cross-cultural validity Internal consistency Test-retest reliability
** **			Jörgensen, S., Forslund, E. B., Lundström, U., Nilsson, E., Richard, L. E. V. I., Berndtsson, E., & Divanoglou, A. (2021). Sound Psychometric properties of the Swedish version of the spinal cord independence measure self-report. Journal of rehabilitation medicine, 53(5).	*n* = 90 Traumatic, non-traumatic or congenital SCI Median age 41 (IQR 22, range 17–74) Traumatic 82%, non-traumatic 17% Tetraplegia 39%, paraplegia 61% Complete injury 42%, incomplete injury 56%	Floor and ceiling effect Internal consistency Measurement error Test-retest reliability
** **			Wang, T., Tang, J., Xie, S., He, X., Wang, Y., Liu, T., … & Li, K. (2021). Translation and validation of the Chinese version of the spinal cord independence measure (SCIM III) self-report. Spinal Cord, 59(10), 1045-1052.	*n* = 147 Complete or incomplete traumatic or non-traumatic SCI Mean age 40.3 (SD 12.9, range 18–65) Traumatic 88.4% Tetraplegia 30.6%, paraplegia 69.4% AIS A 51.8%, AIS B 12.2%, AIS C 21.6%, AIS D 14.4%	Content validity Criterion validity Internal consistency Test-retest reliability
** **			Bonavita, J., Torre, M., China, S., Bressi, F., Bonatti, E., Capirossi, R., … & Scivoletto, G. (2016). Validation of the Italian version of the spinal cord independence measure (SCIM III) self-report. Spinal Cord, 54(7), 553-560.	*n* = 116 Traumatic or non-traumatic SCI Time since injury ≥ 1 month Mean age 45.5 (SD 17.7) Traumaatic 61.2%, non-traumaatic 38.8 % Tetraplegia 43.1%, paraplegia 56.9% AIS A 28.4%, AIS B 13.8%, AIS C 11.2%, AIS D 46.6%	Criterion validity
** **			Tongprasert, S., Wongpakaran, T., & Soonthornthum, C. (2022). Validation of the Thai version of the Spinal Cord Independence Measure Self-Report (SCIM-SR-Thai). Spinal Cord, 60(4), 361-367.	*n* = 61 Traumatic or non-traumatic SCI Mean age 52.2 (SD 15.4) Traumatic 67.2%, non-traumatic 32.8% C1-4 AIS A, B, and C 18.0%, C5-8 AIS A, B, and C 13.1%, T1-S3 AIS A, B, and C 29.5%, AIS D at any injury level 39.4%	Concurrent validity Construct validity Cross-cultural validity Internal consistency
** **			Aguilar-Rodríguez, M., Peña-Pachés, L., Grao-Castellote, C., Torralba-Collados, F., Hervás-Marín, D., & Giner-Pascual, M. (2015). Adaptation and validation of the Spanish self-report version of the Spinal Cord Independence Measure (SCIM III). Spinal Cord, 53(6), 451-454.	*n* = 100 Traumatic or non-traumatic SCI Time since injury ≥ 1 month Mean age 55.4 (SD 15.2) C 19 %, Th 60 %, L 11 %, CE 10 % Complete 33%, incomplete 67% Traumatic 55%, non-traumatic 45%	Concurrent validity Cross-cultural validity

#### CHIEF

One study [59] has assessed the psychometric properties of the CHIEF in a population of people with SCI. Internal consistency was reported to have a Cronbach’s alpha value of 0.92. Test–retest reliability evaluated with intraclass correlation coefficient (ICC) was 0.80 (P < 0.001). The minimal detectable change (MDC) was found to be 0.99, and the standard error of measurement (SEM) was 0.36. Content validity was determined by calculating the content validity ratio (CVR) gained through a rating system of the 25 items by a panel of 10 experts. The formula for calculating the CVR was provided as follows: CVR = (Ne − N/2)/(N/2); (Ne: number of experts rated on item as essential; N: total number of experts in the panel). Content validity index (CVI) was calculated as the mean of the CVR values of the retained items. Excellent content validity was achieved as the CVI value was 0.97. A minimum score of 0.78 is required in CVI for the scale to be rated as having excellent content validity [60].

#### NEFI-SF

So far, one study [40] has been published about the psychometric properties of the NEFI-SF. In a Swiss sample (*n* = 1549), the internal consistency of the NEFI-SF items was reported as Cronbach’s alpha (0.82). The construct validity was analyzed with a partial credit model (a Rasch model for ordinal responses). In consequence, the attitude items concerning colleagues, family, and friends were joined in a testlet. The item fit was good (mean squared errors 0.77–1.22). The mean item difficulty was 0.09, and it was spread along the Rasch scale, but the mean person ability was −1.67. Considering the Rasch estimated item difficulties, a conversion table was created for rescaling the total score in a range from 0 to 100.

A floor effect was found; 23% of the respondents did not report any environmental barriers. These people were more often men, people with incomplete injuries and paraplegia, and they had been injured more recently. There was differential item functioning (DIF) due to completeness of injury in five items: “Public” and “Home access”, “Social attitudes”, “Finances”, and the “Attitudes of close persons” testlet. DIF was caused by the level of SCI for the items of “Finances”, “Personal care assistance” and “Communication devices”. The “Personal care assistance” and “Home access” items were sensitive to the language of the NEFI-SF.

#### SCI-SCS

The psychometric properties of the SCI-SCS have been evaluated in four studies [42, 61–63]. In one study [62], face validity was assessed by a panel of 12 people with SCI. Items were evaluated according to a 10-point Likert scale ranging from 0 (not appropriate) to 10 (strongly appropriate). The Italian SCI-SCS obtained a face validity value of 9.78 out of 10. Cronbach’s alpha values for internal consistency varied from 0.65 to 0.87 in three studies [42, 62, 63]. Arora *et al*. [61] examined inter-rater reliability between the telephone and the paper-pencil administered SCI-SCS, where the ICC was 0.96 (95% confidence interval (CI) 0.93–0.98). The agreement of two telephone-based assessments measure by ICC was 0.90 (95% CI 0.83–0.95). Test–retest reliability was assessed in two studies, where the test–retest reliability coefficient was (*r* = 0.569–0.805) (P < 0.001) [42], and ICC was 0.91 (95% CI 0.78–0.96) [62].

Convergent or concurrent validity were explored in three studies [42, 62, 63]. A summary of their psychometric properties is presented in Table 4. Poor to moderate correlations were found when comparing either the total sum of SCI-SCS to the total score or items of corresponding outcome measures [42, 62, 63] or the single items of SCI-SCS to the items or subsections of other outcome measures [63].

**Table 4:** Summary of convergent and concurrent validity shown as correlations between total score and single items of the Spinal Cord Injury Secondary Conditions Scale (SCI-SCS) and corresponding outcome measures.

SCI-SCS item	Outcome measure	Correlation	P value
Total score	SF-12 Physical functioning items		
	Rating of health	−0.336^a^	0.008 [42]
	Health limited moderate activities	0.359^a^	0.004 [42]
	Health limited climbing several flights of stairs	0.437^a^	< 0.001 [42]
	Accomplished less than would like due to health problems	0.317^a^	0.012 [42]
	Limited in the kind of work or other activities	0.442^a^	< 0.001 [42]
	Degree pain interfered with normal work	0.644^a^	< 0.001 [42]
	How much of the time physical health (and emotional well being) interfered with social activities	0.475^a^	< 0.001 [42]
	EQ VAS	−0.47^a^	< 0.001 [63]
	QoL – General^c^	−0.36^a^	< 0.001 [63]
	QoL – Physical health^c^	−0.36^a^	< 0.001 [63]
	Modified Barthel Index (MBI)	−0.20^b^	0.016 [62]
	Patient health questionnaire 9 (PHQ-9)	0.43^b^	< 0.001 [62]
	General anxiety disorder 7 (GAD-7)	0.30^b^	< 0.001 [62]
	SF-8 Physical component summary	−0.36^b^	< 0.001 [62]
	SF-8 Mental component summary	−0.21^b^	0.014 [62]
Muscle spasms (spasticity)	Spasm frequency scale^d^	0.59^a^	< 0.001 [63]
	Spasm severity scale^d^	0.56^a^	< 0.001 [63]
Chronic pain	Number of pain sites^e^	0.31^a^	< 0.001 [63]
	Level of pain last week^e^	0.45^a^	< 0.001 [63]
Joint and muscle pain	Number of pain sites^e^	0.47^a^	< 0.001 [63]
	Level of pain last week^e^	0.43^a^	< 0.001 [63]

Note: EQ VAS, EuroQol Visual Analogue Scale; SF-8, 8-item Short Form Survey; SF-12, 12-item Short Form Survey.

^a^ Spearman rank correlation.

^b^ Pearson’s *r*.

^c^ International Spinal Cord Injury Quality of Life Basic Data Set.

^d^ Penn Spasm Frequency Scale.

^3^ International Spinal Cord Injury Pain basic dataset 1.0.

Two studies evaluated the construct validity of the SCI-SCS [62, 63]. An explorative factor analysis carried out by Conti *et al*. [62] showed loading for the factors “Genitourinary and bowel”, “Muscle structures and pain”, and “Skin, breathing and metabolism”. A fourth factor, “Circulatory and autonomic”, was not psychometrically robust, but the items it comprises were considered relevant indicators of autonomic cardiovascular dysfunction by the panel of experts involved in the study and therefore maintained in the final version of the instrument. In a confirmatory factor analysis (CFA) by Jørgensen *et al*. [63] four-factor CFA model demonstrated good fit to the data (*χ^2^*, P = 0.056, Comparative fit index (CFI) = 0.918, Root Mean Square Error of Approximation (RMSEA) = 0.035, and Standardized Root Mean Square Residual (SRMR) = 0.050). Factor structure was confirmed for latent factors of “Genitourinary and bowel” and “Muscle structures and pain” but were unable to validate the factors “Skin, breathing and metabolism” and “Circulatory and autonomic”.

There were no floor or ceiling effects present for the total score of the SCI-SCS [63]. In Conti *et al*. [62], the SCI-SCS items were divided into four sub-scales in which floor effect was found in the “Skin, breathing and metabolism” (27%) and “Circulatory and autonomic” (25%) sub-scales.

#### SCIM-SR

Ten studies evaluated the psychometric properties of the SCIM-SR [38, 64–72]. Internal consistency was reported in six studies [66, 67, 69–72]. The Cronbach’s alpha values ranged 0.80–0.98 for the total score, 0.91–0.96 for “Self-care”, 0.51–0.96 for “Respiration and sphincter management”, and 0.84–0.97 for the “Mobility” sub-scales, respectively. In the “Respiration and sphincter management” sub-scale, the item referring to respiration seemed to be a misfit as it appears to assess different constructs than the items referring to sphincter management [67, 69]. Criterion and/or convergent validity was explored in seven studies by comparing the total scores of the SCIM-SR and the Spinal Cord Independence Measure (SCIM III) [38, 64, 65, 69–72]. A summary of the measurement properties is presented in Table 5.

**Table 5:** Summary of the convergent and criterion validity values and ranges of the SCIM-SR.

Convergent and criterion validity					
Correlation between SCIM-SR and SCIM III scores		Mean ranges between SCIM-SR and SCIM III scores		Agreement between SCIM-SR and SCIM III scores	
Pearson correlation coefficient [38,65,70,72]	Range	Bland–Altman plots [38,64,65,69–72]	Range of means	ICC [38,65,70–72]	Range
Total score	*r* = 0.87–0.97	Total score	−0.01–5.14	Total score	0.90–0.95
Self-care	*r* = 0.87–0.96	Self-care	0.06–0.89	Self-care	0.86–0.96
Respiration and sphincter management	*r* = 0.81–0.93	Respiration and sphincter management	0.03–1.56	Respiration and sphincter management	0.76–0.93
Mobility	*r* = 0.87–0.96	Mobility	0.00–3.49	Mobility	0.83–0.97
Spearman's correlation [69]	Value				
Total score	0.95				
Self-care	0.89				
Respiration and sphincter management	0.83				
Mobility	0.89				
Lin's concordance correlation coefficient [64]	Value (95% CI)				
Total score	0.998 (0.997, 0.998)				
Self-care	0.988 (0.982, 0.992)				
Respiration and sphincter management	0.992 (0.988, 0.995)				
Mobility	0.997 (0.995, 0.998)				

Note: CI, Confidence interval. ICC, Intraclass correlation coefficient. SCIM III, Spinal Cord Independence Measure. SCIM-SR, Spinal Cord Independence Measure Self Report.

A floor effect was discovered in one study [72] in which 21.9% of the respondents received the lowest scores in the mobility sub-section. A ceiling effect in the self-care sub-section was present in one study [68] in which 19% and 52% of the two participant-groups achieved the highest scores. Prodinger *et al*. [68] did not find any floor or ceiling effects for the total score of the SCIM-SR.

Test–retest reliability was examined with ICC in two studies [66, 67] where the values ranged 0.97–0.98 for the total score, 0.89–0.96 for “Self-care”, 0.90–0.94 for “Respiration and sphincter management”, and 0.96–0.97 for the “Mobility” sub-scales. Wang *et al*. [71] utilized Spearman’s correlation coefficient to explore the test–retest reliability with correlations of 0.88 for the total score, 0.84 for “Self-care”, 0.74 for “Respiration and sphincter management”, and 0.88 for “Mobility”. Jörgensen *et al*. [66] conducted a Bland–Altman plot analysis where the mean differences for repeated measurements were: total score −0.56 (95% CI −2.33–1.22), limits of agreement (LoA) −7.55–6.43; “Self-care” −0.028 (95% CI −0.38–0.33), LoA −2.10–2.04; “Respiration and sphincter management” −0.50 (95% CI −2.05–1.05), LoA −7.00–6.00; and “Mobility” −0.61 (95% CI −1.51–0.30), LoA −5.63–4.41.

Wilartratsami *et al*. [72] reported the MDC with 95% CI 13.99 for the total score, 4.22 for “Self-care”, 8.34 for “Respiration and sphincter management”, and 5.58 for “Mobility”. In contrast, Jörgensen *et al*. [66] reported the smallest detectable difference of 5.29 for the total score, 1.50 for “Self-care”, 4.96 for “Respiration and sphincter management”, and 3.85 for “Mobility”. Wilartratsami *et al*. [72] reported SEM as 5.05 for the total score, 1.52 for “Self-care”, 3.01 for “Respiration and sphincter management”, and 2.01 for “Mobility”. In the Jörgensen *et al*. [66] study, the SEM for previous domains were 1.91, 0.54, 1.79, and 1.39.

A Rasch-analysis was conducted to examine the construct validity of the SCIM-SR [68]. The initial Rasch analysis with the full sample did not fit the assumptions of the Rasch model. Researchers opted for a testlet design by grouping items into three sub-scales. For a functional differentiation, a sub-grouping was done based on injury completeness and the level of the injury. Following these methods, a fit to the Rasch-model was achieved for the sub-groups of incomplete tetraplegia and complete paraplegia but not for the incomplete paraplegia sub-group (DIF).

A confirmatory factor analysis supported the three-factor structure of the SCIM-SR: CFI 0.928, Tucker-Lewis index (TLI) 0.902, RMSEA 0.101 (90% CI 0.074–1.126), chi-square (*χ^2^*) 200.598, df 126 (*χ^2^*/df = 1.60). However, a uniform structure supported the model better: CFI 0.948, TLI 0.927, RMSEA 0.092 (90% CI 0.061–0.120), *χ^2^* 164.254, df 109 (*χ^2^*/df = 1.50) [70].

## Discussion

To our knowledge, this was the first scoping review to summarize the range and utilization of PROMs in people with SCI from the perspective of the Brief ICF Core Sets for people with SCI. From the 1171 included articles, we identified 595 different relevant PROMs and investigated the frequency of their appearance in the literature. Of the identified PROMs, 55 were reported in ten or more different research articles. Although there are hundreds of different PROMs available, we discovered that only some of them are used frequently. The most frequently used PROMs assess only a limited number of the ICF categories deemed as the most appropriate for describing the health and functioning of people with SCI [18, 20]. Proportionally many studies and applied PROMs focused on measuring either the “Body Functions” domain of the ICF or general health or life satisfaction. Environment and environmental barriers were assessed in studies less often. Additionally, we found an unfortunate number of publications lacking reports of validity and reliability or other solid arguments to support the usage of the selected PROM, a fact also stated in previous reviews [73, 74].

In addition to conventional PROMs, the use of item banks, such as Patient-Reported Outcomes Measurement Information System (PROMIS®) and Spinal Cord Injury – Quality of Life (SCI-QOL) measurement system, was noticeable. PROMIS® and SCI-QOL item banks are collections of items measuring the same domain. From these item banks, fixed or custom forms or single items can be utilized to measure the desired construct [75–77]. Despite the vast usage of PROMIS® and SCI-QOL fixed forms, there was not any one form used frequently enough to fit the inclusion criteria of this study. Traditional PROMs with fixed forms have significant clinical value for their availability and ease of administration. Computer adaptive administration (CAT) of item bank -based PROMs, however, could be a considerable alternative to traditional paper and pen PROMs in the future, should their availability and accessibility increase. As the number of items required in CAT PROMIS® and SCI-QOL are calculated based on previous responses, this method could increase the efficiency of data collection.

Of the PROMs that covered ICF categories of the Brief Core Sets for people with SCI most thoroughly, the psychometry of the SCIM-SR was the most extensively researched. Compared with other PROMs, there were substantially more psychometric studies conducted on the SCIM-SR, and the most measurement properties were assessed according to the COSMIN taxonomy [78]. According to the COSMIN guidelines [33], the SCIM-SR showed sufficient internal consistency for the total score as well as each sub-scale (*α* > 0.70), except for the sub-scale “Respiration and sphincter management” (the lowest alpha value was 0.51). Caution should be exercised when drawing conclusions based on the sub-scale scores as there is some uncertainty about the factor structure of the SCIM-SR [70]. The SCIM-SR demonstrated good test–retest reliability as the ICCs for the total score and each sub-scale were sufficient (ICC > 0.70). In the included studies, the criterion validity was assessed by comparing the SCIM-SR scores to the SCIM III scores, and these comparisons demonstrated sufficient correlations (*r* ≥ 0.70). The validity and reliability of the SCIM III have been previously shown [79–81].

Fewer studies had been carried out to examine the psychometric properties of the remaining selected PROMs. As the developers of the SCI-SCS discussed, further examination of the factor structure is required to progress on the development of this PROM [42]. Evidence of structural validity is currently inconsistent as explorative and confirmatory factor analyses portrayed different results. However, evidence from these two studies supported at least a two-factor structure of the SCI-SCS [62, 63]. Additionally, there is still some uncertainty about the internal consistency of the SCI-SCS as one of the three studies reporting Cronbach’s alpha value failed to meet the desired threshold (*α* > 0.70) [63]. Convergent validity and hypothesis testing of the SCI-SCS had statistically significant but mostly low correlations to other outcome measures, possibly because there are no comparable measures to assess the secondary conditions of SCI [62].

Although the CHIEF and the NEFI-SF appear to be promising PROMs based on the ICF categories covered, there are insufficient psychometric studies conducted on the SCI population. Their measurement properties are unsatisfactory as only one study meeting the inclusion criteria of this review was found on each outcome measure. The CHIEF was not specifically designed to assess environmental barriers among people with SCI, and its psychometric properties have been previously assessed in heterogenous populations [58]. The advantage of the NEFI-SF is that it was tailored to populations with SCI based on the ICF Core Sets for SCI. However, it is a relatively new PROM and lacks evidence of several measurement properties. Therefore, further studies are needed on the psychometric properties of the NEFI-SF.

Our focus was on PROMs that cover the Brief ICF Core Sets for people with SCI most thoroughly. We acknowledge that there are situations where it is desirable to assess a single or a more distinct construct such as pain or depression. Therefore, a psychometrically sound PROM is required. Our scoping review revealed that there has been recent discussion on the utilization of appropriate PROMs for measuring depression [82, 83], bladder and bowel management [84], spasticity [85], and pain [86].

### Strengths and limitations

This scoping review has some limitations. Our eligibility criteria excluded all articles where the PROMs were applied in either telephone or face-to-face interviews. As we also focused on the frequency of the appearance of PROMs in the included articles, data from this scoping review might differ from the true extent and range of the PROM utilization. The reason for our exclusion criteria was that we wanted to study PROM usage where there is no interference from a health care professional [21]. Several studies have also demonstrated discrepancies between the results of self-administered and interviewer-administered PROMs [87–90]. In some studies, such as Blanes *et al*. [91], researchers based their choice of interview-administered PROM on the low literacy rate of the target population. This might cause bias in this scoping review as several articles originating from countries with substantial illiteracy rates were excluded.

In this scoping review, our objective was to identify the most frequently used PROMs. Since we linked only the most prevalent PROMs to the ICF, it is possible that more recently developed PROMs were excluded. However, several studies are required before sufficient information on the psychometrics of a measure can be obtained, and its validity can be demonstrated. Future research should explore the potential of newer PROMs in measuring the desired ICF categories. Assumptions of how frequently PROMs are used were made based on their appearance in published articles. The frequency of PROM utilization in clinical practice might, however, vary. Additionally, our inclusion criteria for the selection of PROMs focused on frequency of appearance in research articles and not the frequency of studies. Instances where there may be multiple published articles from the same study cohort could skew our data for some of the PROMs, although this affected only a small portion of our data.

Out of the most frequently utilized PROMs identified in this review, the number of PROMs behind a paywall was proportionally high. For the results of this scoping review to be beneficial for as many as possible in general clinical practice, we decided to include only PROMs that are freely available. Further research is needed to investigate the non-free PROMs for people with SCI. It should also be noted that our study highlighted the PROMs’ capabilities to measure desired constructs only in the perspective of the Brief ICF Core Sets which does not reveal the full scale of measurable aspects for certain PROMs. Choosing comprehensive ICF Core Sets would have partly helped to include indicators that would have taken better into account issues such as participation.

In this scoping review, all assumptions regarding methods of PROM administration, use of standardized PROMs, data extraction, and data analysis were made solely based on available article publications. We did not contact any authors for supplementary information. Ambiguity and unclearness in reporting the administration and possible modifications of the PROMs used was prevalent, which is consistent with conclusions of a previous systematic review [27]. Additional information from the authors of the screened articles could have confirmed or denied some of the assumptions made in this scoping review.

Due to the scoping review methodology, there was no quality appraisal of the included studies [29, 92]. Therefore, caution is advised when drawing conclusions from our results. Still, the lack of quality evaluation does not affect the frequency of identified PROMs nor their linking to the ICF. It might, however, cause a bias in the summary of psychometric properties of the selected PROMs.

## Conclusions

This scoping review revealed that a wide variety of PROMs are being utilized for people with SCI. However, a majority of the most frequently used and freely available PROMs measure only a limited number of categories out of the Brief ICF Core Sets for people with SCI. After the ICF-linking, four PROMs – the CHIEF, the NEFI-SF, the SCI-SCS and the SCIM-SR – appeared to cover the categories of the Brief Core Sets the most thoroughly. Out of those PROMs the psychometric properties of the SCIM-SR had undergone the most comprehensive examinations. Sufficient evidence of validity and reliability encourages using the SCIM-SR to assess functioning in daily activities of people with SCI. Also, the CHIEF, the NEFI-SF, and the SCI-SCS are promising PROMs in their ability to assess health and functionality according to the ICF. However, additional research is needed on their psychometric properties in people with SCI to form further recommendations. Discussion and international consensus are also needed on whether, and how, valid measuring systems or CAT PROMs should replace traditional individual PROMs, or be applied in conjunction with them, in clinical practice and research.

## Disclaimer statements

**Funding** None.

**Declaration of interest** None.

**Conflicts of interest** Authors have no conflicts of interest to declare.

## Supplementary Material

Supplementary_Table3_List_of_PROMs_extracted.xlsx

Supplementary_Table2_Database_search_SCIM_SR.docx

Supplementary_Table1_Database_search_PROMs.docx

## References

[1] Bickenbach J, Biering-Sørensen F, Knott J, Shakespeare T, Stucki G, Tharion G, Wee J. Understanding spinal cord injury. In: Bickenbach J, Officer A, Shakespeare T, von Groote P, editors. International perspectives on spinal cord injury. Geneva: WHO; 2013. p. 3–10.

[2] Krause JS, Dismuke-Greer CE, Reed KS, Rumrill P. Employment and job benefits among those with spinal cord dysfunction: a comparison of people with spinal cord injury and multiple sclerosis. Arch Phys Med Rehabil. 2019;100(10):1932–1938. 31247166 10.1016/j.apmr.2019.05.031

[3] Bodine C, Burne B, Burns A, Cardenas D, Craven C, Harvey L, Inglis G, Jensen M, Jessup N, Kennedy P, *et al.* Health care and rehabilitation needs. In: Bickenbach J, Officer A, Shakespeare T, von Groote P, editors. International perspectives on spinal cord injury. Geneva: WHO; 2013. p. 68.

[4] Brinkhof M, Al-Khodairy A, Eriks-Hoogland I, Fekete C, Hinrichs T, Hund-Georgiadis M, Meier S, Scheel-Sailer A, Schubert M, *et al.* Health conditions in people with spinal cord injury: contemporary evidence from a population-based community survey in Switzerland. J Rehabil Med. 2016;48(2):197–209. 26931074 10.2340/16501977-2039

[5] Marion TE, Rivers CS, Kurban D, Cheng CL, Fallah N, Batke J, Dvorak MF, Fisher CG, Kwon BK, Noonan VK, *et al.* Previously identified common post-injury adverse events in traumatic spinal cord injury-validation of existing literature and relation to selected potentially modifiable comorbidities: a prospective Canadian cohort study. J Neurotrauma. 2017 Oct 15;34(20):2883–2891. 28562167 10.1089/neu.2016.4933 PMC5653096

[6] Tallqvist S, Kauppila AM, Vainionpää A, Koskinen E, Bergman P, Anttila H, Hämäläinen H, Täckman A, Kallinen M, Arokoski J, *et al.* Prevalence of comorbidities and secondary health conditions among the Finnish population with spinal cord injury. Spinal Cord. 2022 Jul;60(7):618–627. 34511604 10.1038/s41393-021-00704-7 PMC9287167

[7] Rauch A, Cieza A, Stucki G. How to apply the international classification of functioning, disability and health (ICF) for rehabilitation management in clinical practice. Eur J Phys Rehabil Med. 2008 Sep;44(3):329–342. 18762742

[8] Coulter A. Measuring what matters to patients. Br Med J. 2017 Feb 20;356:j816. 28219884 10.1136/bmj.j816

[9] U.S. Department of Health and Human Services FDA Center for Drug Evaluation and Research; U.S. Department of Health and Human Services FDA Center for Biologics Evaluation and Research; U.S. Department of Health and Human Services FDA Center for Devices and Radiological Health. Guidance for industry: patient-reported outcome measures: use in medical product development to support labeling claims: draft guidance. Health Qual Life Outcomes. 2006 Oct 11;4:79. 17034633 10.1186/1477-7525-4-79 PMC1629006

[10] Weldring T, Smith SM. Patient-reported outcomes (PROs) and patient-reported outcome measures (PROMs). Health Serv Insights. 2013 Aug 4;6:61–68. 25114561 10.4137/HSI.S11093 PMC4089835

[11] Kuipers P, Kendall MB, Amsters D, Pershouse K, Schuurs S. Descriptions of community by people with spinal cord injuries: concepts to inform community integration and community rehabilitation. Int J Rehabil Res. 2011 Jun;34(2):167–174. 21490508 10.1097/MRR.0b013e3283460e39

[12] Fellinghauer B, Reinhardt JD, Stucki G, Bickenbach J. Explaining the disability paradox: a cross-sectional analysis of the Swiss general population. BMC Public Health. 2012 Aug 15;12:655. 22894722 10.1186/1471-2458-12-655 PMC3528470

[13] Stucki G, Cieza A, Ewert T, Kostanjsek N, Chatterji S, Ustün TB. Application of the international classification of functioning, disability and health (ICF) in clinical practice. Disabil Rehabil. 2002 Mar 20;24(5):281–282. 12004974 10.1080/09638280110105222

[14] World Health Organization. The international classification of functioning, disability and health (ICF). Geneva: WHO; 2001.

[15] World Health Organization. Towards a common language for functioning, disability, and health: ICF. The international classification of functioning, disability and health. Geneva: WHO; 2002.

[16] Cieza A, Ewert T, Ustün TB, Chatterji S, Kostanjsek N, Stucki G. Development of ICF core sets for patients with chronic conditions. J Rehabil Med. 2004 Jul;44 Suppl:9–11. 10.1080/16501960410015353 15370742

[17] Chen HC, Yen TH, Chang KH, Lin YN, Wang YH, Liou TH, Taiwan ICF Team. Developing an ICF core set for sub-acute stages of spinal cord injury in Taiwan: a preliminary study. Disabil Rehabil. 2015;37(1):51–55. 24597935 10.3109/09638288.2014.895871

[18] Cieza A, Kirchberger I, Biering-Sørensen F, Baumberger M, Charlifue S, Post MW, Campbell R, Kovindha A, Ring H, Sinnott A, *et al.* ICF core sets for individuals with spinal cord injury in the long-term context. Spinal Cord. 2010 Apr;48(4):305–312. 20065984 10.1038/sc.2009.183

[19] Herrmann KH, Kirchberger I, Stucki G, Cieza A. The comprehensive ICF core sets for spinal cord injury from the perspective of physical therapists: a worldwide validation study using the Delphi technique. Spinal Cord. 2011 Apr;49(4):502–514. 21102571 10.1038/sc.2010.155

[20] Kirchberger I, Cieza A, Biering-Sørensen F, Baumberger M, Charlifue S, Post MW, Campbell R, Kovindha A, Ring H, Sinnott A, *et al.* ICF core sets for individuals with spinal cord injury in the early post-acute context. Spinal Cord. 2010 Apr;48(4):297–304. 19786973 10.1038/sc.2009.128

[21] Valkeinen H, Anttila H. The ICF and functional capacity indicators– what, how and why? 2014. Fysioterapia. 2014;4:5–10.

[22] Cieza A, Fayed N, Bickenbach J, Prodinger B. Refinements of the ICF linking rules to strengthen their potential for establishing comparability of health information. Disabil Rehabil. 2019 Mar;41(5):574–583. 26984720 10.3109/09638288.2016.1145258

[23] Meyers AR, Andresen EM, Hagglund KJ. A model of outcomes research: spinal cord injury. Arch Phys Med Rehabil. 2000 Dec;81(12 Suppl 2):S81–S90. 11128907 10.1053/apmr.2000.20629

[24] Scivoletto G, Galli G, Torre M, Molinari M, Pazzaglia M. The overlooked outcome measure for spinal cord injury: use of assistive devices. Front Neurol. 2019 Mar 22;10:272. 30967836 10.3389/fneur.2019.00272 PMC6438886

[25] Churruca K, Pomare C, Ellis LA, Long JC, Henderson SB, Murphy LED, Leahy CJ, *et al.* Patient-reported outcome measures (PROMs): a review of generic and condition-specific measures and a discussion of trends and issues. Health Expect. 2021 Aug;24(4):1015–1024. 33949755 10.1111/hex.13254 PMC8369118

[26] Mokkink LB, de Vet HCW, Prinsen CAC, Patrick DL, Alonso J, Bouter LM, Terwee CB. COSMIN risk of bias checklist for systematic reviews of patient-reported outcome measures. Qual Life Res. 2018 May;27(5):1171–1179. 29260445 10.1007/s11136-017-1765-4 PMC5891552

[27] Whitehurst DG, Engel L, Bryan S. Short form health surveys and related variants in spinal cord injury research: a systematic review. J Spinal Cord Med. 2014 Mar;37(2):128–138. 24559417 10.1179/2045772313Y.0000000159 PMC4066421

[28] Poutanen J, Savolainen S, Shulga A, Arokoski J, Hiekkala S. ICF-linking and psychometric properties of upper extremity mobility outcome measures in spinal cord injury – a scoping review. J Spinal Cord Med. 2024 Mar;47(2):201–213. 36622355 10.1080/10790268.2022.2161867 PMC10885769

[29] Peters MDJ, Godfrey C, McInerney P, Munn Z, Tricco AC, Khalil H. Chapter 11: scoping reviews (2020 version). In: Aromataris E, Munn Z, editors. JBI manual for evidence synthesis, JBI. 2020 [accessed 2022 Dec 4]. https://synthesismanual.jbi.global.

[30] Arksey H, O’Malley L. Scoping studies: towards a methodological framework. Int J Soc Res Methodol. 2005;8(1):19–32.

[31] Tricco AC, Lillie E, Zarin W, O'Brien KK, Colquhoun H, Levac D, Moher D, Peters MDJ, Horsley T, Weeks L, *et al.* PRISMA extension for scoping reviews (PRISMA-ScR): checklist and explanation. Ann Intern Med. 2018 Oct 2;169(7):467–473. 30178033 10.7326/M18-0850

[32] Valkeinen H, Anttila H, Paltamaa J. Opas toimintakyvyn mittarin arviointiin TOIMIA-verkostossa (1.0). [accessed 2023 Mar 8]. https://thl.fi/documents/974257/1449823/Mittariopas_VALMIS_090614+%282%29.pdf/b53595b9-15b8-4fa3-8765-23cd9221de8f.

[33] Prinsen CAC, Mokkink LB, Bouter LM, Alonso J, Patrick DL, de Vet HCW, Terwee CB. COSMIN guideline for systematic reviews of patient-reported outcome measures. Qual Life Res. 2018 May;27(5):1147–1157. 29435801 10.1007/s11136-018-1798-3 PMC5891568

[34] Zigmond AS, Snaith RP. The hospital anxiety and depression scale. Acta Psychiatr Scand. 1983 Jun;67(6):361–370. 6880820 10.1111/j.1600-0447.1983.tb09716.x

[35] Costa P, Perrouin-Verbe B, Colvez A, Didier J, Marquis P, Marrel A, Amarenco G, Espirac B, *et al.* Quality of life in spinal cord injury patients with urinary difficulties. Development and validation of Qualiveen. Eur Urol. 2001 Jan;39(1):107–113. 11173948 10.1159/000052421

[36] Beck AT, Steer RA. Internal consistencies of the original and revised Beck Depression Inventory. J Clin Psychol. 1984 Nov;40(6):1365–1367. 6511949 10.1002/1097-4679(198411)40:6<1365::aid-jclp2270400615>3.0.co;2-d

[37] Ferguson E, Matthews G, Cox T. The Appraisal of Life Events (ALE) scale: reliability and validity. Br J Health Psychol. 1999 May;4(2):97–116.

[38] Fekete C, Eriks-Hoogland I, Baumberger M, Catz A, Itzkovich M, Lüthi H, Post MW, von Elm E, Wyss A, *et al.* Development and validation of a self-report version of the Spinal Cord Independence Measure (SCIM III). Spinal Cord. 2013 Jan;51(1):40–47. 22890418 10.1038/sc.2012.87

[39] Post MW, van der Zee CH, Hennink J, Schafrat CG, Visser-Meily JM, van Berlekom SB. Validity of the Utrecht scale for evaluation of rehabilitation-participation. Disabil Rehabil. 2012;34(6):478–485. 21978031 10.3109/09638288.2011.608148

[40] Ballert CS, Post MW, Brinkhof MW, Reinhardt JD, SwiSCI Study Group. Psychometric properties of the Nottwil Environmental Factors Inventory Short Form. Arch Phys Med Rehabil. 2015 Feb;96(2):233–240. 25264112 10.1016/j.apmr.2014.09.004

[41] Middleton JW, Tate RL, Geraghty TJ. Self-efficacy and spinal cord injury: psychometric properties of a new scale. Rehabil Psychol. 2003;48(4):281–288.

[42] Kalpakjian CZ, Scelza WM, Forchheimer MB, Toussaint LL. Preliminary reliability and validity of a Spinal Cord Injury Secondary Conditions Scale. J Spinal Cord Med. 2007;30(2):131–139. 17591225 10.1080/10790268.2007.11753924 PMC2031942

[43] Widerström-Noga E, Biering-Sørensen F, Bryce T, Cardenas DD, Finnerup NB, Jensen MP, Richards JS, *et al.* The international spinal cord injury pain basic data set. Spinal Cord. 2008 Dec;46(12):818–823. 18521092 10.1038/sc.2008.64

[44] Kroenke K, Spitzer RL, Williams JB. The PHQ-9: validity of a brief depression severity measure. J Gen Intern Med. 2001 Sep;16(9):606–613. 11556941 10.1046/j.1525-1497.2001.016009606.x PMC1495268

[45] Radloff LS. The CES-D scale: a self-report depression scale for research in the general population. Appl Psychol Meas. 1977 Jun;1(3):385–401.

[46] Lovibond SH, Lovibond PF. Manual for the depression anxiety stress scales. 2nd ed. Sydney: The Psychology Foundation of Australia Inc; 1995.

[47] Cohen S, Kamarck T, Mermelstein R. A global measure of perceived stress. J Health Soc Behav. 1983 Dec;24(4):385–396. 6668417

[48] Bouhassira D, Attal N, Alchaar H, Boureau F, Brochet B, Bruxelle J, Cunin G, Fermanian J, Ginies P, Grun-Overdyking A, *et al.* Comparison of pain syndromes associated with nervous or somatic lesions and development of a new neuropathic pain diagnostic questionnaire (DN4). Pain. 2005 Mar;114(1–2):29–36. 15733628 10.1016/j.pain.2004.12.010

[49] Melzack R. The short-form McGill Pain Questionnaire. Pain. 1987 Aug;30(2):191–197. 3670870 10.1016/0304-3959(87)91074-8

[50] Zimet GD, Dahlem NW, Zimet SG, Farley GK. The multidimensional scale of perceived social support. J Pers Assess. 1988;52(1):30–41. 10.1080/00223891.1990.9674095 2280326

[51] Demers L, Weiss-Lambrou R, Ska B. Development of the Quebec user evaluation of satisfaction with assistive technology (QUEST). Assist Technol. 1996;8(1):3–13. 10159726 10.1080/10400435.1996.10132268

[52] Whiteneck GG, Brooks CA, Charlifue S, Gerhart KA, Mellick D, Overholser D, Richardson GN. Guide for use of the CHART. Craig Handicap assessment and reporting technique. 1992.

[53] Fugl-Meyer AR, Bränholm I-B, Fugl-Meyer KS. Happiness and domain-specific life satisfaction in adult northern Swedes. Clin Rehabil. 1991;5(1):25–33.

[54] Kersten P. Impact on participation and autonomy (IPA) manual to the English version: IPA. Southampton: University of Southampton; 2007.

[55] Ware JE. SF-36 health survey. Manual and interpretation guide. Boston: The Health Institute; 1993; 6–1.

[56] Development of the World Health Organization WHOQOL-BREF quality of life assessment. The WHOQOL Group. Psychol Med. 1998 May;28(3):551–558. 9626712 10.1017/s0033291798006667

[57] Krupp LB, LaRocca NG, Muir-Nash J, Steinberg AD. The fatigue severity scale. Application to patients with multiple sclerosis and systemic lupus erythematosus. Arch Neurol. 1989 Oct;46(10):1121–1123. 2803071 10.1001/archneur.1989.00520460115022

[58] Whiteneck GG, Harrison-Felix CL, Mellick DC, Brooks CA, Charlifue SB, Gerhart KA. Quantifying environmental factors: a measure of physical, attitudinal, service, productivity, and policy barriers. Arch Phys Med Rehabil. 2004 Aug;85(8):1324–1335. 15295760 10.1016/j.apmr.2003.09.027

[59] Soni S, Walia S, Noohu MM. Hindi translation and evaluation of psychometric properties of Craig Hospital Inventory of Environmental Factors instrument in spinal cord injury subjects. J Neurosci Rural Pract. 2016 Jan-Mar;7(1):13–22. 26933338 10.4103/0976-3147.172170 PMC4750310

[60] Polit DF, Beck CT. The content validity index: are you sure you know what's being reported? Critique and recommendations. Res Nurs Health. 2006 Oct;29(5):489–497. 16977646 10.1002/nur.20147

[61] Arora M, Harvey LA, Lavrencic L, Bowden JL, Nier L, Glinsky JV, Hayes AJ, *et al.* A telephone-based version of the spinal cord injury-secondary conditions scale: a reliability and validity study. Spinal Cord. 2016 May;54(5):402–405. 26193815 10.1038/sc.2015.119

[62] Conti A, Clari M, Arese S, Bandini B, Cavallaro L, Mozzone S, Vellone E, Frigerio S, *et al.* Validation and psychometric evaluation of the Italian version of the Spinal Cord Injury Secondary Conditions Scale. Spinal Cord. 2020 Apr;58(4):496–503. 31745247 10.1038/s41393-019-0384-z

[63] Jørgensen V, von Rosen P, Butler Forslund E. Considerations on the psychometric properties and validity of the Spinal Cord Injury Secondary Conditions Scale. Spinal Cord. 2021 Aug;59(8):894–901. 34172927 10.1038/s41393-021-00655-z

[64] Aguilar-Rodríguez M, Peña-Pachés L, Grao-Castellote C, Torralba-Collados F, Hervás-Marín D, Giner-Pascual M. Adaptation and validation of the Spanish self-report version of the Spinal Cord Independence Measure (SCIM III). Spinal Cord. 2015 Jun;53(6):451–454. 25510190 10.1038/sc.2014.225

[65] Bonavita J, Torre M, China S, Bressi F, Bonatti E, Capirossi R, Tiberti S, Olivi S, Musumeci G, Maietti E, *et al.* Validation of the Italian version of the Spinal Cord Independence Measure (SCIM III) Self-Report. Spinal Cord. 2016 Jul;54(7):553–560. 26481705 10.1038/sc.2015.187

[66] Jörgensen S, Butler Forslund E, Lundström U, Nilsson E, Levi R, Berndtsson E, Divanoglou A. Sound psychometric properties of the Swedish version of the Spinal Cord Independence Measure Self-Report. J Rehabil Med. 2021 May 28;53(5):jrm00197. 33948671 10.2340/16501977-2839 PMC8814884

[67] Khatri P, Prasertsukdee S, Suttiwong J. Reliability of the Nepali version of the Spinal Cord Independence Measure Self-Report. Zakaria MN, editor. Rehabil Res Pract. 2022 Jun 9;2022:1–7. 10.1155/2022/9983464 PMC9203215 35720259

[68] Prodinger B, Ballert CS, Brinkhof MW, Tennant A, Post MW. Metric properties of the Spinal Cord Independence Measure – Self Report in a community survey. J Rehabil Med. 2016 Feb;48(2):149–164. 26926919 10.2340/16501977-2059

[69] Takeuchi S, Uemura O, Unai K, Liu M. Adaptation and validation of the Japanese version of the Spinal Cord Independence Measure (SCIM III) Self-Report. Spinal Cord. 2021 Oct;59(10):1096–1103. 33931747 10.1038/s41393-021-00633-5

[70] Tongprasert S, Wongpakaran T, Soonthornthum C. Validation of the Thai version of the Spinal Cord Independence Measure Self-Report (SCIM-SR-Thai). Spinal Cord. 2022 Apr;60(4):361–367. 35228652 10.1038/s41393-022-00779-w

[71] Wang T, Tang J, Xie S, He X, Wang Y, Liu T, Jia M, *et al.* Translation and validation of the Chinese version of the Spinal Cord Independence Measure (SCIM III) Self-Report. Spinal Cord. 2021 Oct;59(10):1045–1052. 33446937 10.1038/s41393-020-00601-5

[72] Wilartratsami S, Luksanapruksa P, Santipas B, Thanasomboonpan N, Kulprasutdilok P, Chavasiri S, Chavasiri C. Cross-cultural adaptation and psychometric testing of the Thai version of the Spinal Cord Independence Measure III-Self Report. Spinal Cord. 2021 Mar;59(3):291–297. 32963364 10.1038/s41393-020-00556-7

[73] Jensen M, Stoelb B, Molton I. Measuring pain in persons with spinal cord injury. Top Spinal Cord Inj Rehabil. 2007 Oct;13(2):20–34.

[74] Magasi SR, Heinemann AW, Whiteneck GG. Quality of life/participation committee. Participation following traumatic spinal cord injury: an evidence-based review for research. J Spinal Cord Med. 2008;31(2):145–156. 18581661 10.1080/10790268.2008.11760705 PMC2565477

[75] Cella D, Choi SW, Condon DM, Schalet B, Hays RD, Rothrock NE, Yount S, Cook KF, Gershon RC, Amtmann D, *et al.* PROMIS® adult health profiles: efficient short-form measures of seven health domains. Value Health. 2019 May;22(5):537–544. 31104731 10.1016/j.jval.2019.02.004 PMC7201383

[76] Hays RD, Bjorner JB, Revicki DA, Spritzer KL, Cella D. Development of physical and mental health summary scores from the patient-reported outcomes measurement information system (PROMIS) global items. Qual Life Res. 2009 Sep;18(7):873–880. 19543809 10.1007/s11136-009-9496-9 PMC2724630

[77] Tulsky DS, Kisala PA, Victorson D, Tate DG, Heinemann AW, Charlifue S, Kirshblum SC, Fyffe D, Gershon R, Spungen AM, *et al.* Overview of the Spinal Cord Injury–Quality of Life (SCI-QOL) measurement system. J Spinal Cord Med. 2015 May;38(3):257–269. 26010962 10.1179/2045772315Y.0000000023 PMC4445018

[78] Mokkink LB, Terwee CB, Patrick DL, Alonso J, Stratford PW, Knol DL, Bouter LM, *et al.* The COSMIN study reached international consensus on taxonomy, terminology, and definitions of measurement properties for health-related patient-reported outcomes. J Clin Epidemiol. 2010 Jul;63(7):737–745. 20494804 10.1016/j.jclinepi.2010.02.006

[79] Bluvshtein V, Front L, Itzkovich M, Aidinoff E, Gelernter I, Hart J, Biering-Soerensen F, Weeks C, Laramee MT, Craven C, *et al.* SCIM III is reliable and valid in a separate analysis for traumatic spinal cord lesions. Spinal Cord. 2011 Feb;49(2):292–296. 20820178 10.1038/sc.2010.111

[80] Glass CA, Tesio L, Itzkovich M, Soni BM, Silva P, Mecci M, Chadwick R, el Masry W, Osman A, Savic G, *et al.* Spinal Cord Independence Measure, version III: applicability to the UK spinal cord injured population. J Rehabil Med. 2009 Sep;41(9):723–728. 19774305 10.2340/16501977-0398

[81] Itzkovich M, Gelernter I, Biering-Sorensen F, Weeks C, Laramee MT, Craven BC, Tonack M, Hitzig SL, Glaser E, Zeilig G, *et al.* The Spinal Cord Independence Measure (SCIM) version III: reliability and validity in a multi-center international study. Disabil Rehabil. 2007 Dec 30;29(24):1926–1933. 17852230 10.1080/09638280601046302

[82] Placeres AF, Fiorati RC. Assessment instruments and depression rates in people with spinal cord injury: a systematic review. Rev Esc Enferm USP. 2018 Dec 13;52:e03388. 30570079 10.1590/S1980-220X2017037303388

[83] Titman R, Liang J, Craven BC. Diagnostic accuracy and feasibility of depression screening in spinal cord injury: a systematic review. J Spinal Cord Med. 2019 Oct;42(sup1):99–107. 31573447 10.1080/10790268.2019.1606556 PMC6781470

[84] Tate DG, Wheeler T, Lane GI, Forchheimer M, Anderson KD, Biering-Sorensen F, Cameron AP, Santacruz BG, Jakeman LB, Kennelly MJ, *et al.* Recommendations for evaluation of neurogenic bladder and bowel dysfunction after spinal cord injury and/or disease. J Spinal Cord Med. 2020 Mar;43(2):141–164. 32105586 10.1080/10790268.2019.1706033 PMC7054930

[85] Ertzgaard P, Nene A, Kiekens C, Burns AS. A review and evaluation of patient-reported outcome measures for spasticity in persons with spinal cord damage: recommendations from the ability network – an international initiative. J Spinal Cord Med. 2020 Nov;43(6):813–823. 30758270 10.1080/10790268.2019.1575533 PMC7808317

[86] Sawatzky B, Bishop CM, Miller WC, SCIRE Research Team. Classification and measurement of pain in the spinal cord-injured population. Spinal Cord. 2008 Jan;46(1):2–10. 17968403 10.1038/sj.sc.3102137

[87] Meyers AR, Andresen EM. Enabling our instruments: accommodation, universal design, and access to participation in research. Arch Phys Med Rehabil. 2000 Dec;81(12 Suppl 2):S5–S9. 11128904 10.1053/apmr.2000.20618

[88] Erhart M, Wetzel RM, Krügel A, Ravens-Sieberer U. Effects of phone versus mail survey methods on the measurement of health-related quality of life and emotional and behavioural problems in adolescents. BMC Public Health. 2009 Dec 30;9:491. 20042099 10.1186/1471-2458-9-491 PMC2809066

[89] Caute A, Northcott S, Clarkson L, Pring T, Hilari K. Does mode of administration affect health-related quality-of-life outcomes after stroke? Int J Speech Lang Pathol. 2012 Aug;14(4):329–337. 22472032 10.3109/17549507.2012.663789

[90] Kisala PA, Boulton AJ, Cohen ML, Slavin MD, Jette AM, Charlifue S, Hanks R, Mulcahey MJ, Cella D, *et al.* Interviewer- versus self-administration of PROMIS® measures for adults with traumatic injury. Health Psychol. 2019 May;38(5):435–444. 31045427 10.1037/hea0000685 PMC6506178

[91] Blanes L, Carmagnani MI, Ferreira LM. Quality of life and self-esteem of persons with paraplegia living in São Paulo, Brazil. Qual Life Res. 2009 Feb;18(1):15–21. 18989756 10.1007/s11136-008-9411-9

[92] Grant MJ, Booth A. A typology of reviews: an analysis of 14 review types and associated methodologies. Health Info Libr J. 2009 Jun;26(2):91–108. 19490148 10.1111/j.1471-1842.2009.00848.x

