# Supplementary material for: Patient-Reported Outcome Measures for assessing functioning, disability, and health of people with spinal cord injury – a scoping review
Source: J Spinal Cord Med. 2025 May 5;49(2):220–40. doi: 10.1080/10790268.2025.2477364 (PMC12931359; doi:10.1080/10790268.2025.2477364)
Supplement: Supplementary_Table2_Database_search_SCIM_SR.docx [file YSCM_A_2477364_SM7768.docx]

Supplementary table 2. Database search example for the psychometric properties of the SCIM-SR

Ovid MEDLINE(R) and Epub Ahead of Print, In-Process, In-Data-Review & Other Non-Indexed Citations and Daily <1946 to August 04, 2023>

1 ("statistical property" or "statistical properties" or psychometric* or clinimetr* or clinometr* or "measures (instruments)" or "Clinical Assessment Tools Evaluation" or "Clinical Assessment Tool Evaluation" or validation or validity or reliability or feasibility or "validation study" or "validation studies" or "feasibility study" or "feasibility studies" or generalizability or generalisability or objectivity or neutrality or trustworthiness or applicability or transferability or interpretability or consistency or consistencies or stability or reproducib* or replica* or repeatab* or sensitivity or specificity or precision or imprecision or concordance or predictive or predictab* or repeatability or responsiveness or instrument or instruments or instrumentation or "test construction" or construct or unidimensional* or dimensionality or inter-rater or intra-rater or intrarater or intratester or "intra-tester" or intertester* or "inter-tester" or intraobserver* or "intra-observer" or interobserver* or "inter-observer" or "intra-examiner*" or intraexaminer* or "inter-examiner*" or interexaminer* or interindividual* or "inter-individual*" or intraindividual* or "intra-individual*" or intra-assay* or interassay* or "inter-assay*" or "minimal detectable" or "minimally detectable" or "minimum detectable" or "minimal important" or "minimally important" or "minimal real" or "minimally real" or "minimal clinically important difference" or "minimal clinically important differences" or "clinically significant change" or "clinically significant changes" or "meaningful change" or "meaningful changes" or "real change" or "real changes" or "smallest change" or "smallest changes" or "detectable change" or "detectable changes" or "detectable difference" or "detectable differences" or discriminative or discriminant or "observer variation" or "observer variations" or "test retest" or "test-retest" or retest* or "precise value" or "precise values" or "standard error" or "standard errors" or "standard deviation" or "standard deviations" or "mean square error" or "mean square errors" or "mean square deviation" or "mean square deviations" or "error of measurement" or "errors of measurement" or "measurement error" or "measurement errors" or "measurement uncertainty" or "measurements uncertainty" or "measurement of uncertainty" or "measurements of uncertainty" or "uncertainty measure" or "uncertainty measures" or "uncertainty in measurement" or "uncertainty in measurements" or responsiveness or responsivity or "item response" or "item responses" or "item selection" or "item selections" or "item correlation" or "item correlations" or "item reduction" or "item reductions" or variability or variance or invariance or covariance or "item functioning" or "item response" or "item responses" or "computer adaptive test" or "computer adaptive tests" or "computer adaptive testing" or "intraclass correlation" or "intraclass correlations" or "interscale correlation" or "interscale correlations" or "factor structure" or "factor structures" or "factorial structure" or "factorial structures" or "factor analyse" or "factor analysis" or "Factor extraction" or "Factor extractions" or coefficient* or "known group" or "known groups" or "multitrait scaling analyse" or "multitrait scaling analysis" or "multitrait multimethod" or equivalence or "cross-cultural comparison" or "cross-cultural comparisons" or "ceiling effect" or "ceiling effects" or "floor effect" or "floor effects" or kappa* or alpha* or Rasch or Cronbach* or "goodness of fit" or "goodness-of-fit").ab,hw,ti. 7169559

2 "Spinal Cord Independence Measure- Self Report".ab,hw,ti. 16

3 "SCIM-SR".ab,hw,ti. 15

4 2 or 3 21

5 1 and 4 13
