# Supplementary material for: Patient-Reported Outcome Measures for assessing functioning, disability, and health of people with spinal cord injury – a scoping review
Source: J Spinal Cord Med. 2025 May 5;49(2):220–40. doi: 10.1080/10790268.2025.2477364 (PMC12931359; doi:10.1080/10790268.2025.2477364)
Supplement: Supplementary_Table1_Database_search_PROMs.docx [file YSCM_A_2477364_SM7767.docx]

Supplementary table 1. Database search example for relevant patient-reported outcome measures

Ovid MEDLINE(R) and Epub Ahead of Print, In-Process, In-Data-Review & Other Non-Indexed Citations and Daily <1946 to December 13, 2022>

1 Spinal Cord Injuries/ 43184

2 Paraplegia/ 13133

3 Quadriplegia/ 8365

4 sci.mp. 40736

5 Spinal Injuries/ 9116

6 "spinal cord injur*".mp. 57320

7 "spinal injur*".mp. 14356

8 tetrapleg*.mp. 4820

9 parapleg*.mp. 23554

10 quadripleg*.mp. 10850

11 1 or 2 or 3 or 4 or 5 or 6 or 7 or 8 or 9 or 10 111902

12 Patient Reported Outcome Measures/ 12641

13 "Surveys and Questionnaires"/ 549993

14 Self Report/ 41778

15 Diagnostic Self Evaluation/ 4060

16 Self-Assessment/ 13254

17 "patient-reported outcome measur*".mp. 19836

18 prom?.mp. 10450

19 "self-assessment?".mp. 25455

20 survey?.mp. 1174846

21 questionnaire?.mp. 913244

22 "self report*".mp. 208115

23 "self-evaluation*".mp. 9870

24 12 or 13 or 14 or 15 or 16 or 17 or 18 or 19 or 20 or 21 or 22 or 23 1591030

25 11 and 24 5436

26 25 and "Humans".sa_suba. 4743

27 limit 26 to english language 4539
